# Personal

**Published:** 1903-04

**Authors:** 


### PERSONAL.

DR. WENDELL C. PHILLIPS, of New York, has removed from 350 Madison Avenue to 40 West 47th Street. Hours: 10-1.

---

DR. JOHN H. DANIELS, of Buffalo, has removed from 434 Ashland Avenue to 559 West Ferry Street. Hours: morning until 9; afternoon, 1 to 3; evening, 7 to 8. Both telephones.

---

PROFESSOR G. N. CALKINS, who fills the chair of zoology at Columbia University, New York, lectured at the Buffalo University Medical College, Tuesday, March 17, 1903, at 5 p.m. His subject was Metabolism of the cell and protoplasma of old age. It was a highly entertaining and instructive delineation of a series of studies the lecturer has been conducting.

---

DR. HERBERT MICKLE has removed from Buffalo to Cleveland, O. Dr. Mickle leaves many friends in this city who will regret the change he has felt constrained to make.

---

DR. BURT C. JOHNSON, of Buffalo, sailed for Europe March 21, 1903, to spend a few months in study at educational centers in the old world.

---

DR. HARRY MEAD, of Buffalo, has gone abroad for study in the capitals of Europe. He sailed March 21, 1903, on the Hamburg-American liner "Blucher."

Dr. George Ryerson Fowler, of Brooklyn, surgeon with the rank of colonel, who has lately served on the staff of Major-General Roe, has been made a brigadier-general by brevet in recognition of meritorious service in the National Guard for more than twenty-five years.

---

Dr. John McGaw Woodbury, the commissioner of street cleaning in New York, has been appointed surgeon of the 22d Infantry, N. Y., N. G., vice Surgeon Bennett S. Beach, retired. Dr. Woodbury has proved the most efficient executive of the department of street cleaning that New York has ever had, not excepting Col. Waring. The past winter has witnessed an unusual number of snow blockades in New York, but Dr. Woodbury has cleared the streets with surprising promptitude and marvelous skill.

---

Dr. Walter D. Greene, health commissioner of Buffalo, has been appointed a state medical inspector, to serve as representative of the state department of health during the prevalence of typhoid fever at West Seneca.

---

Dr. Edward Clark, deputy commissioner of health of Buffalo, has received an appointment similar to that of Dr. Greene, as stated above, and for like reasons and purposes.

---

Dr. E. H. Ballou, health officer of West Seneca, has been giving close attention to the typhoid fever epidemic at West Seneca and states that he regards it now under control. He has had the coöperation of Drs. Greene and Clark, as representatives of the state department of health. It has been a large undertaking in view of the pollution of Smokes Creek and great credit is due Dr. Ballou for the efficient service he has rendered.

---

Dr. Richard H. Satterlee, of Buffalo, recently entertained the Gross Medical Club in his apartments at The Touraine. The club membership comprises physicians residing in and near Buffalo, most of whom were present with their wives on this occasion, the ladies being entertained by Mrs. Satterlee. Dr. J. D. MacPherson, of Akron, read a paper entitled, Popular medical education; its influence in limiting the spread of disease, which will be published in a future number of the Journal.

Dr. Chauncey Pelton Smith, of Buffalo, has been appointed surgeon of the Buffalo, Rochester, and Pittsburg railroad.

Dr. L. J. McAdam, of Buffalo, has removed from 315 Jersey Street to 441 Dearborn Street.

